# *A Ticket to Ride*: The Implications of Direct Intercellular Communication *via* Tunneling Nanotubes in Peritoneal and Other Invasive Malignancies

**DOI:** 10.3389/fonc.2020.559548

**Published:** 2020-11-26

**Authors:** Emil Lou

**Affiliations:** Department of Medicine, Division of Hematology, Oncology and Transplantation, University of Minnesota, Minneapolis, MN, United States

**Keywords:** ovarian cancer, mesothelioma, tunneling nanotubes, tumor microtubes, intercellular communication, confocal microscopy, peritoneal cancers, cancer pathophysiology

## Abstract

It is well established that the role of the tumor microenvironment (TME) in cancer progression and therapeutic resistance is crucial, but many of the underlying mechanisms are still being elucidated. Even with better understanding of molecular oncology and identification of genomic drivers of these processes, there has been a relative lag in identifying and appreciating the cellular drivers of both invasion and resistance. Intercellular communication is a vital process that unifies and synchronizes the diverse components of the tumoral infrastructure. Elucidation of the role of extracellular vesicles (EVs) over the past decade has cast a brighter light on this field. And yet even with this advance, in addition to diffusible soluble factor-mediated paracrine and endocrine cell communication as well as EVs, additional niches of intratumoral communication are filled by other modes of intercellular transfer. Tunneling nanotubes (TNTs), tumor microtubes (TMs), and other similar intercellular channels are long filamentous actin-based cellular conduits (in most epithelial cancer cell types, ~15-500 µm in length; 50–1000+ nm in width). They extend and form direct connections between distant cells, serving as conduits for direct intercellular transfer of cell cargo, such as mitochondria, exosomes, and microRNAs; however, many of their functional roles in mediating tumor growth remain unknown. These conduits literally create a physical bridge to create a syncytial network of dispersed cells amidst the intercellular stroma-rich matrix. Emerging evidence suggests that they provide a cellular mechanism for induction and emergence of drug resistance and contribute to increased invasive and metastatic potential. They have been imaged *in vitro* and also *in vivo* and *ex vivo* in tumors from human patients as well as animal models, thus not only proving their existence in the TME, but opening further speculation about their exact role in the dynamic niche of tumor ecosystems. TNT cellular networks are upregulated between cancer and stromal cells under hypoxic and other conditions of physiologic and metabolic stress. Furthermore, they can connect malignant cells to benign cells, including vascular endothelial cells. The field of investigation of TNT-mediated tumor-stromal, and tumor-tumor, cell-cell communication is gaining momentum. The mixture of conditions in the microenvironment exemplified by hypoxia-induced ovarian cancer TNTs playing a crucial role in tumor growth, as just one example, is a potential avenue of investigation that will uncover their role in relation to other known factors, including EVs. If the role of cancer heterocellular signaling *via* TNTs in the TME is proven to be crucial, then disrupting formation and maintenance of TNTs represents a novel therapeutic approach for ovarian and other similarly invasive peritoneal cancers.

## Introduction

The tumor microenvironment (TME) is lush with a complex array of interacting parts. These parts include a multitude of cell types that evolve during the course of carcinogenesis and cancer cell progression. Some components with the highest metastatic potential splinter off and metastasize to other TME niches in the body. Peritoneal cancers, such as primary ovarian cancer and peritoneal mesotheliomas, are characterized by a hallmark of localized or regional dissemination within the peritoneal cavity. Many of these cancers can be challenging to treat clinically in advanced stages, and the underlying biology of invasion remains an active area of investigation due to lack of full understanding of the cellular biology driving this process. Ovarian cancer in particular is one of many cancers in which the role of the TME in cancer progression is relatively well-established. The ovarian TME not only comprises immune infiltration and angiogenesis, but also unique features that are characterized by regional metastasis within the peritoneum. A recent comprehensive article by Worzfeld et al. published in this journal discussed in detail the extreme molecular and cellular diversity of the ovarian cancer microenvironment (1); this diversity presents a complex set of problems when it comes to translational application of knowledge of the ovarian TME and tumoral reaction in the face of biological insults stimulated by chemotherapeutic drugs and surgical resections.

Much of the focus of cell-cell communication in the TME of ovarian and other peritoneal malignancies such as malignant pleural or peritoneal mesothelioma has focused on gap junction-mediated cellular transfer and nearly exclusively on extracellular vesicles (EVs) (1). This focus mirrors many other articles focusing on this rapidly expanding and exciting field of cancer cell biology. However, excessively narrow focus on EVs risks ignoring other similarly exciting developments in the field of intercellular communication that may prove equally vital in the dynamics TME. One of these alternate models of communication is a form of long, thin membranous conduits arising from filamentous actin-based cellular extensions, a class that includes a number of types that have been variably designated as tunneling nanotubes (TNTs) (2–33), tumor microtubes (TMs) (34–37), and other names in the published literature to date.

Here, let us examine ovarian and other peritoneal malignancies as paradigms for understanding the niche of TNTs in the TME, and their potential role in instigating cancer cell invasion, metastasis, and drug resistance. Ovarian cancer is one of the deadliest cancers in women, with a high rate of emergence of chemoresistant disease. Approximately, 240,000 new cases of ovarian cancer are diagnosed worldwide, and approximately 65% of these women die of the disease each year (38). The peritoneum is the most common site for dissemination of ovarian cancer, and about two-thirds of women present with advanced stage disease (39). Standard-of-care treatment for ovarian cancer is platinum-based chemotherapy after debulking surgery, when feasible. While most ovarian cancers fully respond to initial first-line chemotherapy, the majority of advanced-stage ovarian cancers develop chemoresistance. In addition, 20%–30% of patients with ovarian cancer have platinum-resistant disease. Unfortunately, recurrent ovarian cancers are rarely curable. Innovative research is needed to identify the biologic causes of the emergence of chemoresistance and new therapeutic targets for treating women with platinum-resistant disease. Much of the current landscape over the past decade, as has been the case with most solid tumor malignancies, has zeroed in on identification of actionable genomic alterations for improving patient outcomes. However, the full canvas of the TME remains less understood, but is no less elaborate and important as a focus of investigation. This point is especially true as therapeutic targeting of genomic drivers has inevitably led to cellular mutations that counteract these strategic approaches. To date, there are few reports of the role of TNTs in ovarian cancer biology (8, 22, 23, 30, 40); thus, this review will incorporate and cite pertinent findings in the field uncovered in additional cellular cancer models that are similarly invasive and difficult-to-treat in human patients, with the premise that at least some findings are universal and will also apply globally to TNTs in peritoneal cancers.

A hallmark of the advanced ovarian cancer TME is the process of tumor angiogenesis (41, 42). Angiogenesis is a prototypical process for tumor-stromal interactions, leading clinically to highly invasive and ultimately chemoresistant and metastatic tumors in patients. The stromal components of tumors are heterogeneous and include cancer-associated fibroblasts, immune cells, and also endothelial cells that comprise the tumor vasculature. Advances in understanding the vascular endothelial growth factor (VEGF) pathway of molecular signaling have allowed targeting this pathway with kinase inhibitors or immunotherapy approaches (e.g., bevacizumab); these anti-VEGF therapies have led to significant increases in progression-free survival (PFS) for a number of VEGF-rich forms of cancer, including ovarian cancer (43–45). However, the use of current drugs targeting angiogenesis has not resulted in increases in overall survival for patients with advanced ovarian tumors. For example, bevacizumab plus chemotherapy was used in clinical trials for patients with platinum-sensitive recurrent ovarian cancer; although addition of bevacizumab improved PFS by 4 months, it did not improve overall survival (45, 46). Nonetheless, as manifested clinically by increased PFS, it was clear that inhibition of angiogenesis had some biologic activity; this is an excellent example of a potential “clinic-to-lab bench” research path that can bear fruit by identifying elements of the TME that contribute to angiogenesis, generating knowledge that can then be used to improve future therapeutic strategies targeting this aspect of the TME more effectively in clinical trials.

## Enter the Matrix: Potential Roles of TNTs in the Heterogeneous Tumor Ecosystem

**Figure 1:**
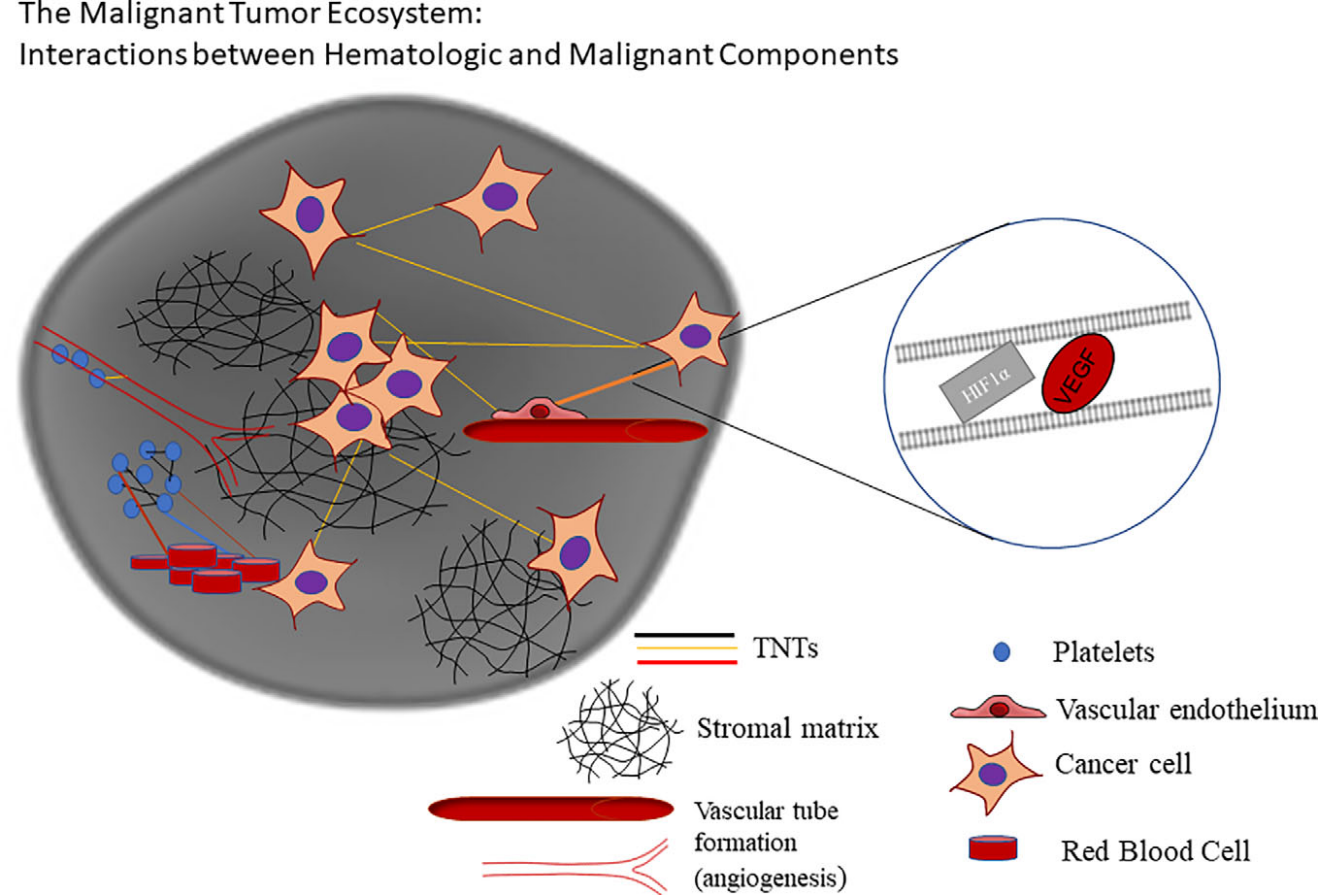
Schematic of dynamic interplay and cross-talk via hypoxia-induced TNTs facilitating intercellular transfer of cargo implicated in angiogenesis and other processes critical to the tumor ecosystem (adapted from a figure previously published in Lou et al., *Frontiers in Cellular and Developmental Biology*, October 2018, and available for use here per Creative Commons BY 4.0 guidelines).

The above biologic background provides opportunities for clarifying the role of long-range cell-cell communication *via* TNTs in the complex TME. TNTs are a heterogeneous group of ultrafine actin-based cellular protrusions that are distinct from other protrusions in that they serve as direct channels for intercellular communication in the TME. Their diversity extends to the size of their ultrastructure, with reports of TNTs spanning 15–500 µm in length across some epithelial and mesenchymal cancer cell types, and widths spanning 50–1000+ nm (4, 35, 47, 48). Since the first designation of these structures as “TNTs” more than a decade and a half ago (49), subsequent studies have elaborated on findings that these and similar forms of channeled protrusions do not always adhere to this arbitrary set of measurements, including a class more recently designated as “tumor microtubes” (TMs) in glioblastoma in *in vivo* animal models and others (34–36). A common factor of this growing family of actin-based extensions is that they can extend and form direct connections between distant cells, or form between adjacent or nearly adjacent cells moving apart, to function as direct open-ended conduits for direct intercellular transfer of cellular cargo, such as (but not limited to) mitochondria, exosomes, or microRNAs (23, 29, 30, 50). TNTs have been imaged in intact tumors using confocal microscopy, demonstrating their potential *in vivo* relevance (23, 35, 47). Investigators in this burgeoning field have proposed that TNTs/TMs provide a physical and previously missing link in the chain of active players in cell communication in the dynamic and rapidly evolving TME, in ovarian as well as many other forms of similarly invasive malignancy. This physical connection is not limited to cancer-cancer cell interactions, but also can embrace a higher level of all-cell communication that is otherwise coined “tumor-stromal” or “heterotypic” cell interactions, including among the cell types described above—vascular endothelium, immune cells, reactive mesothelium in the inflammation-rich peritoneum of ovarian cancer patients, and more (**Figure 1**).

Timing of cellular activity is an underlooked, but no less crucial, aspect of TME evolution. How cells act in the changing TME, and react to external stimuli *via* intercellular communication *via* any means, will inevitably affect response or lack of response to therapy. The role of players in cell communication in this 4D approach are not entirely clear, but conceivably will be clarified with advancements of technologies such as *in vivo* imaging (e.g., intravital microscopy) that has been used to visualized EVs (51, 52). Using tumor specimens resected from patients, or grown and examined *ex vivo* from animal models, the extent of immune infiltration of solid tumor in general can now be clarified and quantified in more detail than ever before. In ovarian carcinoma, diverse immune cell populations that include tumor-associated macrophages, natural killer (NK) cells, and dendritic cells regulate immune inhibition vs. activation, and thus heavily impact prognosis (53). Some of the early publications and also recent work in the field of TNTs focused specifically on TNT function following formation between immune cells, including NK cells, macrophages, and dendritic cells (14, 54–59). While most of these studies were evaluated in non-cancer microenvironments, and despite some of these reported nanotubes differing in their physical structure and extent of connectivity compared to cancer cell TNTs, these studies nonetheless cumulatively provide background for TNT-mediated immune cell-tumor cell mutual cooperation and communication in the TME. T lymphocytes are an additional immune cell type shown to form TNTs *via* Fas-regulated signaling (60, 61). The success of T-cell targeting immuno-oncologic strategies in solid tumor malignancies—specifically targeting programmed cell death protein 1 (PD1) and its ligand PDL1, or cytotoxic T lymphocyte associated antigen 4 (CTLA4)—has brought great attention to intracellular pathways triggered by these targets (62). While there is no clear association between these proteins and TNT formation or function as of yet, these pathways provide an additional point of convergence for immuno-oncology, tumor-immune cell interface by infiltration, and the role of TNTs in both.

## Peritoneal Cancers, Including Ovarian Carcinoma and Malignant Mesothelioma, are Prime Models for Investigation of TNTs in Cancer

**Figure 2:**
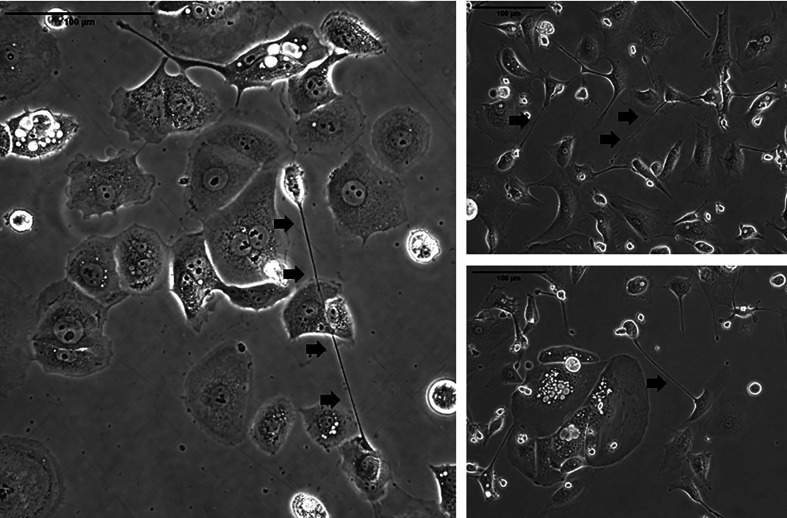
Representative examples of TNTs (indicated along their length by arrowheads) connecting ovarian carcinoma cells in culture. The images on the left are of cells from the OvCar 3 cell line. The two images on the right are of cells derived from a malignant effusion (ascites) fluid from women with advanced ovarian malignancy. Scale bars = 100 μm.

TNTs reproducibly form between ovarian cancer cells in co-culture (**Figure 2**). Physical characteristics that differentiate these unique actin-based protrusions include the fact that they are relatively thinner and longer than other protrusions such as filopodia, invadopodia, etc.; however, their most notable characteristic that distinguishes them visually is their lack of adherence to the substratum when cultured *in vitro*. The limitations of 2D cell culture are well known; thus, we and others have examined TNTs using 3D culture techniques, including use of Matrigel, collagen, and fibronectin matrices (4). An area of great research interest is to establish whether TNT-mediated intercellular communication between ovarian cancer cells and tumor stromal cells facilitates tumor growth in recurrent ovarian cancer models. We have published early work supporting the notion that ovarian cancer TNTs contribute to hypoxia-induced angiogenesis by activating endothelial cells in the TME (63), and also reported that expression of hypoxia inducible factor-1α (HIF-1α) was 2-fold higher in platinum-resistant ovarian cancer cells than platinum-sensitive cells when cultured in hypoxic conditions (8). Collectively, these findings provide some basis for the role of TNTs in mediating tumor angiogenesis in recurrent ovarian cancer, and further investigation is clearly required to follow up on these observations.

**Figure 3:**
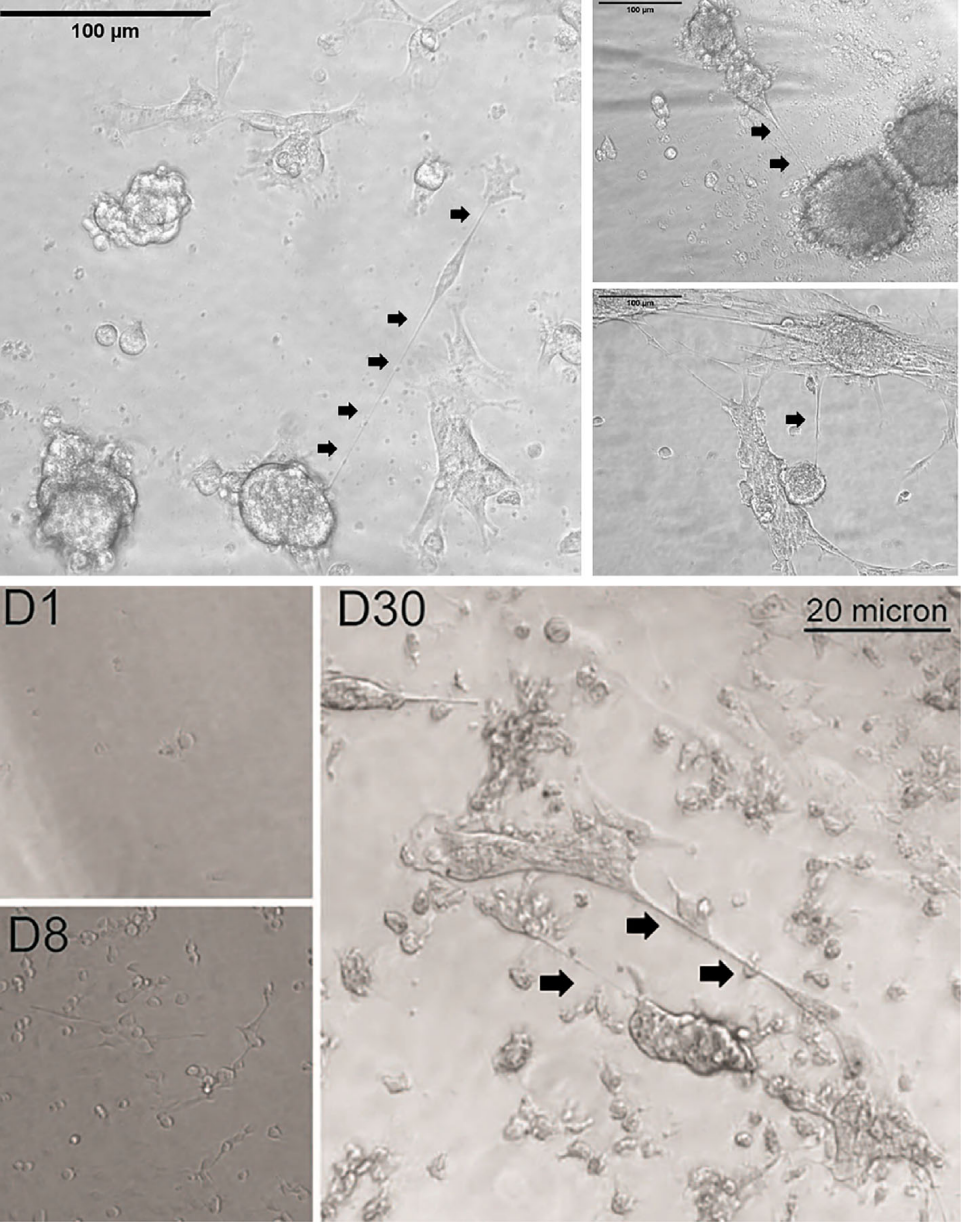
Examples of TNTs connecting individual cells, as well as clusters of cells (single-cell clone-derived spheroids), from malignant pleural mesothelioma. As cells divided and proliferated across the available space, the non-adjacent cells simultaneously formed TNTs that acted as tethers, forming a large and vast syncytial network of these cancer cells. In the top set of images, VAMT (sarcomatoid malignant pleural mesothelioma) cells were cultured in a clonogenic dilution assay under non-adherent conditions. By 24 (left) and 29 (right) days, a single cell proliferated and formed spheroids connected by TNTs as shown. Scale bars = 100 μm. The bottom set of images demonstrates progression from a single or small number of single VAMT cells (e.g., D8 = Day 8), through 30 days of cell culture (D30) at which time a large aggregate of cells is connected by TNTs and TMs to other clusters of cells. Scale bar = 20 μm.

Hallmarks of peritoneal cancers include compartmental inflammation that is extremely conducive to tumor-stromal remodeling, which in turn facilitates regional spread of these tumors (64). Mesothelial surfaces that include the visceral, serosal, and parietal layers of abdominal organs or the walls of the abdomen and pelvis provide physical scaffolds for locally invasive growth of these cancers. This advancement takes the form of tumor nodules and also an accumulation of intraperitoneal fluid (ascites) that forms a primordial source of a cytokine-rich inflammatory “soup” that is ripe for providing an environment that stimulates cancer cell proliferation and other alteration of the actin-based cytoskeletal structures. In this context, the physical link to high invasive potential and chemoresistance may be TNTs. The ability of TNTs to weave a web of cellular syncytial networks by connecting many—perhaps dozens or even hundreds of peritoneal cancer cells—is a distinct possibility because TNTs can form between multiple cells. Metastatic ovarian carcinoma within the peritoneum serves as a prime clinical example of a model cancer in which this creation would be impactful on cell persistence, localized invasion, and recurrence. This cancer is particularly resilient despite multimodality treatment (e.g., surgical debulking and chemotherapy, even considering historical or more recent tactics for intraperitoneally administered chemotherapeutic drugs as well as those given systemically). Malignant pleural or peritoneal mesotheliomas are similarly invasive at the local level. We have shown that, when cultured *in vitro* under non-adherent conditions, these cells can indeed proliferate and either singly, or as aggregates or as single-cell derived (clonal) spheroids. These grouped cells are then capable of forming TNTs to interconnect vast numbers of cells, not unlike nodular clusters of cells that form peritoneal studs that are sizeable enough to be visualized on laparoscopy and potentially on radiologic imaging as well. Examples of this concept are provided in **Figure 3**. This finding has potential implications for efficacy of regional therapeutic strategies, such as intraperitoneal administration of chemotherapy in women with advanced epithelial ovarian carcinomas (65). One example of the potential importance of TNT-mediated cell tethering of spheroids is that it would facilitate direct TNT-specific cell-to-cell communication in the evolution of resistance to this treatment, in a more specific manner than EVs can perform in this fluid-filled environment.

## TNTs and Links to Emergence of Chemoresistance in These Cancers: An Evolving Story

**Figure 4:**
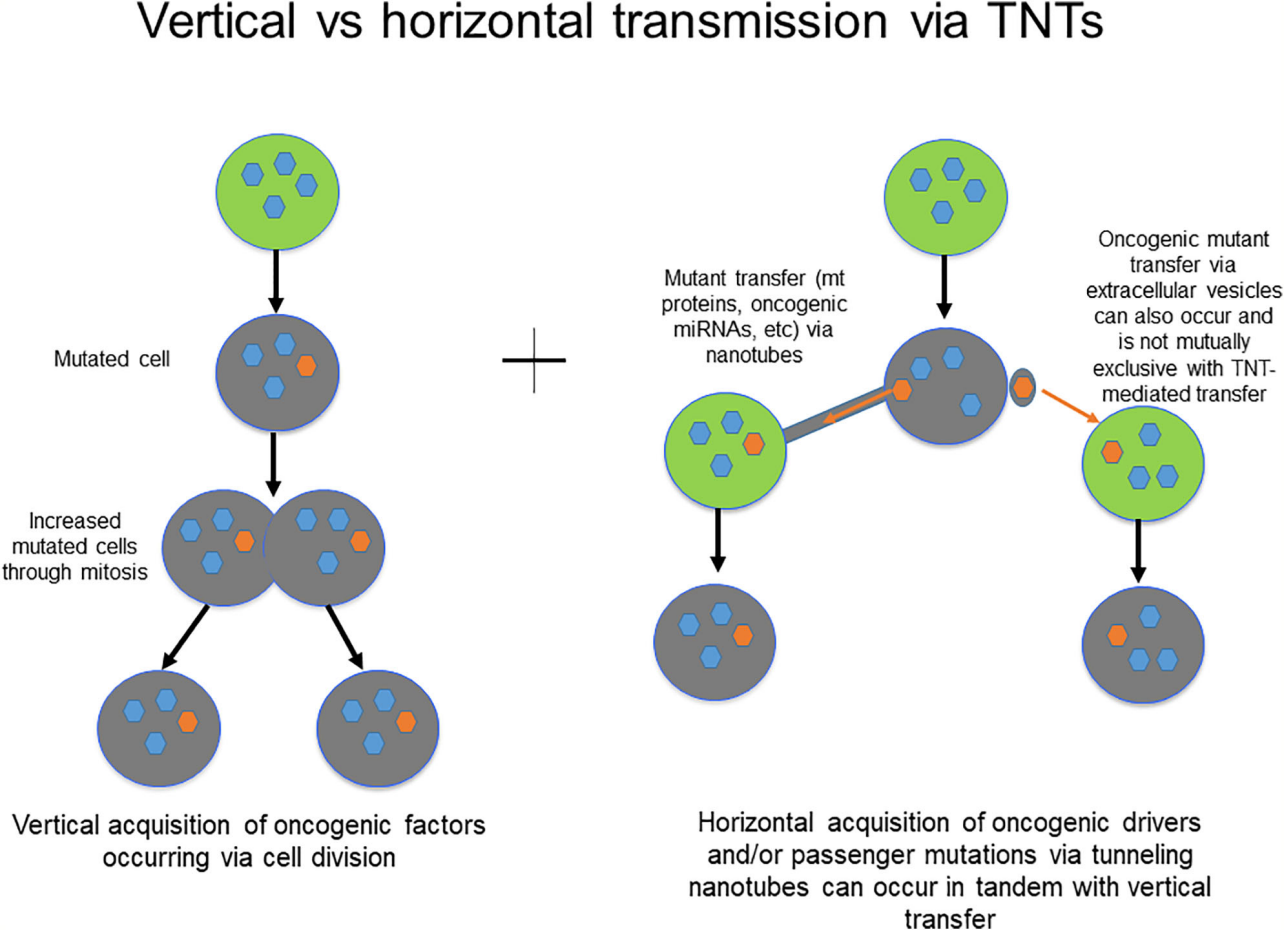
Schematic demonstrating the principles of vertical vs. horizontal transmission of molecular messengers, and potential role for TNTs/TMs in mediating the latter process.

Could TNTs be potential predictive and/or prognostic biomarkers in peritoneal cancers? The concept of TNTs as conduits of cell communication in the TME, and the role of horizontal communication in general, as crucial mediators of tumor invasion and therapeutic resistance is still evolving. While the current body of data is relatively limited, it is reasonable to expect that within the coming decade, more specific roles of TNTs in this niche will be elucidated. This information will lead to enhanced understanding of how these roles are distinct from well-established alternate forms of communication, including gap junctions and EVs in particular. Current paradigms accept the idea that chemoresistance emerges as a result of mutations in key regulatory genes with cells passing these genetic mutations *via* vertical transmission to daughter cells through mitotic division and clonal expansion. However, horizontal (cell-to-cell) transmission of regulatory factors *via* channels of cellular communication including TNTs could also be responsible for the development of chemotherapy resistance (**Figure 4**).

This concept is not wholly new, however. In fact, models of direct cell-to-cell transfer as a method of development of chemoresistance in ovarian cancer were suggested as far back as 30 years ago (66, 67). New discoveries in channels of communication during the past 10+ years—prominently including the discovery and improved characterization of TNTs—have opened the door to renewing investigation of this potential mechanism of chemoresistance. A News Feature in *Nature* in September 2017, and additional coverage Quanta Magazine and Scientific American a year later, highlighted landmark advances in this field and effectively marked the field of TNT research in diseases such as cancer as an emerging field of cell biology. With an increasing critical mass of researchers from backgrounds inclusive of basic cell biology, cancer cell biology, and molecular biology, and other researchers from outside the cancer research spectrum applying their knowledge to this growing field, it is inevitable that the body of data will grow in the next several years.

The approach to focusing on highly relevant clinical problems from a cellular (rather than solely molecular) oncology focus is unique in its approach. The intersection of hypoxia, angiogenesis, cell-cell communication, in general, and even more specifically, the communication that is occurring *via* TNTs, is discussed in this article. This is an example of strategic approach for addressing a well-known clinical problem (recurrence and chemoresistance of ovarian cancer) by exploring how all of these factors together can facilitate the evolution of the TME to induce chemoresistance. Ultimately, it may be determined that this evolution takes place at the cellular rather than exclusively at the molecular level, and that confirmation would open the space of cellular oncology from a more translational perspective. What would truly mark a point of maturation of the field of TNT biology is accurate identification of molecular and structural biomarkers that can help distinguish accurate identification of TNTs from other cellular structures and most especially from other forms of cellular protrusions. Identifying specific biomarkers of TNTs in cancer, and in disease states in general, is a Holy Grail of this field.

## Identifying TNT-Specific Biomarker Genes in Ovarian and Other Cancers: A Next Frontier and Challenge in the Field of TNT Biology

Some promising studies to date have identified factors upregulated or at least associated with TNTs, TMs, and other TNT-like structures in cells of neuronal and immune cell origin, for example, but thus far there are no validated markers that correlate with presence of TNTs in ovarian or similar cancers. Nonetheless, some promising strides have been made in that direction in the past decade. Pasquier et al. have examined heterocellular TNT connections between malignant and stromal cells as modulators of ovarian cancer chemoresistance. This acquisition of resistant properties was associated with mitochondrial transfer and also transmission of membrane p-glycoprotein, whose expression has long been associated with drug efflux activity into the extracellular space (22, 23).

TNT cellular networks are upregulated between cells (including cancer cells and between tumor and stromal cells) under conditions of physiologic and metabolic stress, including exposure to hydrogen peroxide, serum deprivation, and hyperglycemia (47, 68, 69); this finding implicates the potential role of TNTs in facilitating tumor-stromal interactions in the complex, dynamic, and ever-changing heterogeneous tumor microenvironment. Conditions that support and otherwise stimulate these forms of stress in the TME, and molecular pathways that are hyperactivated under these conditions, may induce or accelerate formation of TNTs. mTOR is one example for TNT formation in ovarian cancer.

An example of a potential marker and critical factor in these protrusions was provided by Jung et al. in an orthotopic model of glioblastoma. Utilizing gene expression microarrays, they identified candidate genes including tweety-homologue 1 (TTYH1), and associated its presence with the ability to regulate TM-mediated cancer cell invasion and proliferation, whereas downregulation nearly abrogated this effect (34). Additional strategic approaches using drug screens have also yielded insight into potential molecular pathways, and individual members of pathway cascades, that are potentially vital to TNTs.

Suppression of TNT formation by targeting the mTOR pathway either directly with everolimus, or indirectly with metformin, demonstrated that TNT suppression negatively regulates intercellular transfer, marking mTOR and related downstream players as potential targets for disrupting TNT function in ovarian carcinomas (8) and mesotheliomas (47). However, in this case, as in the field in general, TNT/TM-based studies have been performed across a wide spectrum of cell types. As a result, it is not yet clear to what degree some findings are specific to TNTs across the cell spectrum, or whether some findings are cell type-specific. Advances in larger scale screening and genomics may aid in answering this and similar questions in more rapid fashion in the future from a molecular perspective.

Malignant pleural mesothelioma (MPM) is another paradigm for cancers that are highly invasive locally and also refractory to various forms of treatment. MPM has also proven to be a highly useful model for studying TNTs, as cell lines and cells directly derived from human patients readily form TNTs when cultured *in vitro*. Findings in MPM also serve as a model for research approach in ovarian cancer, as these two cancers have a similarly invasive fashion. Our own experience in genetic screening of MPM cells cultured in conditions that upregulate TNTs demonstrated differences in expression of genes associated with cancer cell invasion and some associated with TNT formation in other, non-cancerous, cell types specifically [Reference: Figure 9, Ady et al., *Frontiers in Physiology*, 2014 (4)]. Investigating relative differences in gene expression between these cells cultured in passage medium vs. culture conditions, we found increased TNT formation in low-serum, hyperglycemic conditions. We quantitated RNA levels of M-Sec (which is also called TNFaip2, or tumor necrosis factor-α-induced protein 2) and transmembrane MHC Class III leukocyte specific transcript 1 (LST1), two gene products that are known to be enriched in TNTs (70, 71). Both genes were significantly upregulated in the mesothelioma cells cultured in TNT-inducing medium compared to control medium. While literature searches of both M-Sec/TNFaip2 or LST1 do not crossmatch with any published studies in ovarian carcinoma specifically, studies of LST1 in bladder cancer cells point to it playing a vital role in TNT formation in these cells through interaction with the Ras-independent Guanine Nucleotide Exchange Factor RalGPS2 (72). In turn, RalGPS2 is a HIF-2α-induced gene directly involved in endothelial cell sprouting (73) (and thus angiogenesis) under hypoxic conditions that upregulate TNT formation in ovarian cancer cells (8). Studies in separate cell lines derived from cervical cancer (HeLa) and osteosarcoma (U2-OS) further implicated LST1 in TNT formation through promoting interaction of exocyst complexes with RalA GTPase (71). While similar studies have not been specifically reported in ovarian carcinoma to date, there is opportunity to extrapolate work done in other such cancer models to ovarian cancer models for further investigation.

When we examined key genes involved in cellular migration and invasion in mesothelioma, we found that cells grown in TNT medium all significantly upregulated expression of tenascin-C, CD44, osteopontin, fascin, and mesothelin. These data confirmed detectable differences in adaptive gene expression profiles leading to TNT formation and a higher malignant potential, and serve as one example of strategic approach for confirming or identifying biomarkers that, even if not specific to TNTs, are at least upregulated in these forms of cell protrusions.

## Nanotubes and Their Role in Creating Intratumoral Heterogeneity and Facilitating Cancer Chemoresistance: The Road to Treatment Perdition?

Hypoxic conditions stimulate angiogenesis in solid tumor malignancies. The role of intercellular communication in expediting this process remains unclear. Intratumor heterogeneity (ITH) likely plays a larger role than previously given credit for. Furthermore, while the existence of disparate subclonal populations creating a ITH state in ovarian and other carcinomas is well known, how the state of ITH evolves and comes about has been less well-studied. One important consideration is that stromal, non-malignant cells comprise as much as 80%–90% of a given tumor’s volume; in fact, higher proportion of stroma has been associated with worse prognosis in invasive solid tumor malignancies (74–81). In 2019, our group reported its finding that tumor-stroma proportion assessed from specimens at diagnosis also correlates with eventual emergence of platinum chemotherapeutic drug resistance in women with ovarian carcinoma (82). At the individual cell and multicellular levels, the actual population of malignant cells that compose only 10%–20% or more of the tumor may not be in close enough proximity to exchange cellular information *via* gap junctions. Thus, there is opportunity for TNTs and similar modes of long-to-medium range communication to, literally, bridge that gap and mediate cell-to-cell interaction.

The above postulate holds for interactions among malignant cells, but the ability of TNTs to bridge distant cells is not limited only to malignant cell communication. TNTs may also facilitate the early stages of angiogenesis through long-range connection of malignant and vascular endothelial cells and consequent exchange of cellular signals that are important to angiogenesis. Co-cultures of vascular endothelium and malignant cells has confirmed that intercellular connections can reproducibly form *via* TNTs *in vitro* (63). Acceleration of this process in a hypoxic stroma-rich ovarian TME would create optimal conditions that drive molecular machinery known to be associated with chemoresistance. At the same time, it produces a niche for TNTs to form in more abundance than other conditions, paving the way for them to stimulate a cellular network of exchange of oncogenic and chemoresistance-driving factors.

The identity of such chemoresistance-driving factors can be widely variable. Arguably, the best-studied cargo of TNT transfer to date has been organelle transfer, quite specifically mitochondria. Pasquier et al.’s study discussed earlier associated TNT-mediated intercellular transfer of mitochondria and p-glycoprotein with chemoresistance in ovarian and breast carcinoma cells (22, 23). microRNAs, as well as siRNAs, as examples of genetic material that can also be cargoed between cancer cells *via* TNTs (30, 83), as an arguably much more direct and efficient route than has been reported in studies showing EV-mediated transfer including in ovarian cancer specifically (84–86). Our study evaluating intercellular trafficking of oncogenic microRNAs (miRNAs) differentially expressed in ovarian cancer cells associated with platinum chemoresistance, compared to drug-sensitive cells, included visualization of this horizontal transfer between malignant ovarian cells as well as between cancer-stroma (e.g., malignant ovarian <-> benign ovarian epithelium) (30). Importantly, this transport was not limited as one might have expected to transport from chemoresistant to chemosensitive cells, or more generally from malignant to benign; rather, trafficking was bi-directional. An emerging question of interest, informed by use of advancing microscopy techniques including Cryo-electron microscopy, focused ion beam scanning electron microscopy (FIB-SEM), and others, is whether the heterogeneity of cellular protrusions that over time have been coined as singular nanotubes and microtubes, are in fact “multi-lane cellular highways” represented by multiple tubes running in parallel (27, 63). This has been nicely illustrated in work from the Zurzolo lab at the Institute Pasteur published in 2019; this report provided insight into thin individual TNTs (iTNTs) that together may comprise the highways that taken together provide an ability to mediate bi-directional rather than only uni-directional trafficking of cargo (27). Overall, all of these findings opened new possibilities into identifying TNTs as a potential therapeutic target through a novel strategy: disrupting horizontal cell-to-cell transfer of miRNAs, or other cargo, that stimulate increased cellular invasive capabilities and chemoresistance.

## TNTs and Similar Channels of Communication in the TME: What’s the Physiological Relevance?

Knowledge of TNT biology has increased especially rapidly during the last several years, progressing from questions about its relevance and provability, to forward-thinking applicability to human diseases such as cancer. What has emerged is a clearer picture of TNTs as a universal phenomenon (not tissue or cell-type specific) that is exacerbated and upregulated under conditions that favor disease states. As already mentioned, TNTs are capable of mediating transfer of a wide variety of potential cellular cargo, including those that are known to transfer between cells *via* other means (e.g., miRNA exchange *via* exosomes). To use an example of potential translational relevance to cancer, cell-contact dependent intercellular exchange of the oncogene RAS between immune cells (T lymphocytes, NK cells) was demonstrated over a dozen years ago (87). Such a finding has wide-ranging implication in modern understanding of the role of immune infiltration in the evolving and chemoresistance tumor microenvironment. In 2019, we reported oncogenic KRAS transfer between malignant colon cancer cells as well (10); collectively, these findings are only the beginning of opening a door to understanding the role of TNT-specific communication on the landscape of intratumoral heterogeneity, immune cell-cancer cell interactions, and other heterotypic as well as homotypic interactions and downstream effects. This notion may be most pertinent to cancers in which RAS alterations are most prevalent (e.g., pancreatic and colorectal carcinomas); although KRAS is only mutated in <1% of high-grade serous ovarian carcinomas, mutations in KRAS or BRAF and other components of the mitogen-activated protein kinase (MAPK) pathway are more prevalent in low-grade serous and also serous borderline forms of ovarian tumors (88–90). Regardless, this finding that oncogenes and/or oncogenic proteins can be acquired *via* horizontal exchange *via* TNTs may thus also have relevance to gynecologic malignancies. Overall, the concept of cell transfer of oncogenic products may also have overarching effects across all forms of cancer, regardless of specific cancer type-specific drivers.

In addition to the above considerations, there is also understanding that there may be considerable heterogeneity among TNTs themselves, including in their physical characteristics (e.g., variability in TNT length and width), timeliness (e.g., TNTs are most likely to form when cells are located at longer distances), and prevalence across cell types (e.g., highly prevalent in cancer compared with non-cancer cell types). There are multiple aspects contributing to diversity of TNTs, including structural components. Gap junctions, composed of connexin-lined channels, have been well-established as a mode of communication between adjacent cells. Some studies have reported connexins located at the tips of TNTs, such as in (non-malignant) astrocytes (32), whereas other studies have found variability that may be cell type-dependent. Regarding the intersection of connexins/gap junctions and TNTs, we have previously discussed this in the context of molecular networking of cells connected by TNTs, and evaluation of mesothelioma found an inverse relation of connexin expression to TNT formation in this model (63). In this case, when expressed, connexins localized to the base and tips of TNTs, supporting their role and presence may be selective but nonetheless part of this mix (63). Beyond just presence, the presence of specific connexin channel subtypes—specifically Connexin 43 (Cx43) has been reported to facilitate intercellular distribution of calcium *via* TMs (35). The role of connexin expression in cancer cells, in relations to both TNTs and EVs, remains to be elaborated upon, and also determined whether other isoforms of connexins including Cx43 play a necessary and sufficient role in TNT/TM formation and function.

In addition to the aforementioned structural heterogeneity of TNTs and TMs, there is just as much diversity in their duration. This diversity may be dependent on cell type, and in the context of cancer, perhaps even the underlying metabolic machinery and site of origin (i.e., organ and TME) of any given cancer. Further differences may be explained by the fact that most of the work to date in this field has been performed in standard two-dimensional cell culture conditions, which may not be fully representative or accurately simulate physical conditions of the 3-D TME. While the current body of *in vivo* data to date is quite limited, TMs in an orthotopic animal model of glioblastoma have been shown to last hours and even days (35). By contrast, studies evaluating duration of cancer cell TNTs have reported that they may last minutes to hours; our team’s earliest studies of TNTs in malignant mesothelioma, for example, utilized time-lapse microscopy to demonstrate that TNTs connecting MPM could last 4–6 h, and in many cases, this was ultimately limited due to physical conditions of cell crowding and also by cell motility (47). The influence of intercellular communication *via* TNTs and TMs may in fact be longer lasting, beyond their presence, and this angle is another unique one that merits investigation in this field.

Work in the field to date has established a rationale for investigating TNTs in depth because they are upregulated in the most aggressive forms of cancer, and their function includes facilitating transport of materials that enhance carcinogenesis and/or chemoresistance. We and others have identified TNTs and similar cell protrusions in the intact tumor architecture of ovarian tumors (8, 23) and other tumor types from human patients (83, 91, 92), yet beyond qualitative description of these findings, as a field we still do not know the full biological implication and activity of TNTs *in vivo*. In the short-term, the results from this work will shed light on the cellular interactions that lead to ineffective therapy in women with advanced ovarian cancers. The tour de force set of studies from the Winkler lab in Heidelberg, Germany have provided strong data in an *in vivo* animal tumor model that TNTs and TNT-like structures induce resistance to radiation therapy (34–37). Examples like this one provide a growing foundation of evidence that, indeed, TNTs/TMs transcend identification as just a physical cellular structure and indeed they do have physiological relevance to cancer and implications for efficacy of therapy.

## Exploiting the TNT and TM Cellular Networking Phenomenon for Intratumoral Drug Distribution

Hijacking naturally occurring heterotypic formation of TNTs between stromal cells and malignant cells in ovarian cancer is also under investigation as a novel therapeutic strategy. Guo et al. utilized antitumor macrophages loaded with the alkylating chemotherapeutic agent doxorubicin as a “Trojan horse” to enhance entry of the drug into target cancer cells (40). In doing so, they uncovered TNT networking as a mechanism by which these cells efficiently distributed the drug to more cells, in effect amplifying the initial effects of doxorubicin and creating a chemotherapeutic equivalent of the bystander effect seen in viral oncolytic infection (93). This finding is similar in nature to our own work demonstrating intercellular distribution of a GFP-expressing engineered therapeutic form of herpes simplex-virus, whose oncolytic effect was amplified following administration of the nucleoside analog ganciclovir due to cell-to-cell spread of viral thymidine kinase *via* TNTs (3). As with Guo et al., we visualized direct intercellular transport of autofluorescent doxorubicin *via* TNTs connecting ovarian carcinoma cells; this transport resulted in apoptosis of the recipient cell (9). Ultimately, whether cancer-targeting cargo being transmitted between cells *via* this mechanism consists of a chemotherapeutic or biologic drug, whether encapsulated or not, or takes the form of biologic agents such as therapeutic viral vectors, there is ample opportunity to create a novel niche of drug delivery through examination of TNTs and TMs in this space.

## Ongoing and Future Directions: How the Field of TNT Biology in Peritoneal and Other Cancers Will Emerge From Obscurity

In the long-term, the work done to date and ongoing studies from a growing number of researchers around the world will provide new directions for drug screens and successful treatment tactics for patients suffering from ovarian as well as other deadly forms of peritoneal and others forms of cancer. This progress will also move the field of cell-cell communication forward in these cancers as a model for aggressive and chemoresistant forms of cancers as a whole. In addition, although still relatively new, the field of TNT biology has begun to emerge from obscurity over the past 5 years with more data demonstrating their role in tumor invasiveness and chemotherapeutic resistance in aggressive tumors *in vivo* (35–37, 94, 95). New validated experimental assays and approaches will be crucial for standardizing the evaluation of TNTs in other model systems as well, and for maximizing reproducibility across labs. The concepts discussed here were formulated from a translational context and from an understanding of the important role of hypoxia in tumor advancement, and based on published work from our group that TNT formation is upregulated in hypoxic settings. Further, this formation is abrogated by drugs that suppress TNT formation when ovarian cancer cells are under normoxic conditions, but not in hypoxia (8). Ultimate identification of TNT-specific biomarkers will enable efforts designed to effectively investigate TNTs more legitimately as viable therapeutic targets. If TNTs mediate intercellular transfer of chemoresistant factors and/or contribute to tumor “molecular networking” between cancer cells and endothelial cells in the tumor matrix, then selectively disrupting or “cutting off” lines of TNT communication will represent an important and highly innovative therapeutic strategy.

## Author Contributions

The author confirms being the sole contributor of this work and has approved it for publication.

## Funding

The author would like to thank the following sponsors of research work in this field in the Lou Lab: several patients with cancer and/or friends and family in honor of patients with cancer treated at the Masonic Cancer Center, Minneapolis, Minnesota; the Minnesota Ovarian Cancer Alliance; The Randy Shaver Cancer Research and Community Fund; the Litman Family Fund for Cancer Research; the Mu Sigma Chapter of the Phi Gamma Delta Fraternity, University of Minnesota; and the American Association for Cancer Research (2019 AACR-Novocure Tumor-Treating Fields Research Grant, grant number 1-60-62-LOU).

## Conflict of Interest

The author declares that the research was conducted in the absence of any commercial or financial relationships that could be construed as a potential conflict of interest.
